# Cultural Humility in Action: Learning From Refugee and Migrant Women and Healthcare Providers to Improve Maternal Health Services in Australia

**DOI:** 10.1111/hex.70106

**Published:** 2024-11-21

**Authors:** Delia Rambaldini‐Gooding, Katarzyna Olcoń, Luke Molloy, Leissa Pitts, Sofia Lema, Eman Baghdadi, Jane Williams, Chris Degeling

**Affiliations:** ^1^ University of Wollongong Wollongong New South Wales Australia; ^2^ Multicultural and Refugee Health Service Illawarra Shoalhaven Local Health District Wollongong New South Wales Australia

**Keywords:** Cultural humility, Health policy, Maternal health, Migrant women, Refugee women, Service providers

## Abstract

**Introduction:**

Access to culturally appropriate healthcare is vital to ensure refugee and migrant women receive optimal care, particularly during the perinatal period. Refugee and migrant women report lower satisfaction with pregnancy care due to language barriers and a perceived lack of understanding of their needs. The aim of this study is to explore how to improve the experiences of migrant and refugee women with maternal health services through the lens of cultural humility.

**Methods:**

Working collaboratively with maternal health service providers and managers and local refugee and migrant women, this research project used a World Café methodology to provide these stakeholders with an opportunity to discuss maternal healthcare in the region. World Café participants (*n* = 34) included women from multicultural backgrounds (*n* = 20), maternal healthcare providers such as midwives, social workers and management (*n* = 5) multicultural healthcare providers (*n* = 7) and a community‐based birth educator (*n* = 1). Data were analysed thematically.

**Results:**

A key finding of the World Café was the need for staff training that is co‐designed and co‐delivered with members of multicultural communities and healthcare providers to enhance the practice of cultural humility. Training should focus on women's stories that capture the cultural nuances around pregnancy and birthing, their support needs including trauma‐informed care, and the importance of effective cross‐cultural communication.

**Conclusion:**

This research gave refugee and migrant women a voice in future decision‐making, specifically in maternal health staff training. The refugee and migrant women shared their perspectives on how to enhance cultural humility practices in maternity services for them. The research has led to opportunities such as community‐based antenatal classes and improvements in maternity services development strategies.

**Public Contribution:**

The project actively engaged with maternal healthcare providers, multicultural and refugee healthcare providers and women from multicultural communities in the design of the project and as participants. Their expertise and experience have been invaluable and have informed pilot programmes that emerged from this study.

## 1 Introduction

Access to culturally appropriate healthcare is vital to ensure refugee and migrant women receive optimal services, particularly during the perinatal period [1]. We use the term ‘refugee and migrant women’ to refer to women who come from non‐English speaking backgrounds who have migrated to Australia to reside, including those seeking asylum [2]. Women from these groups are a key focus within Government policy in Australia, as they experience significant barriers to accessing health services [3], poorer pregnancy outcomes [4, 5] and lower satisfaction with pregnancy care [1, 6]. Recent research indicates that attempts by various levels of the Australian Government [7, 8] to address these issues have not resulted in culturally responsive maternal healthcare [9] and gaps between policy and practice continue. The reason for this is a lack of understanding among practitioners about what the enactment of cultural humility looks like in practice and a limited attempt by healthcare organisations to rethink how they conceptualise it.

As an approach to practice, *cultural humility* has been gaining importance worldwide in shaping equitable health and social services [10, 11]. Cultural humility is centred around respect and openness to the cultural beliefs and practices of people receiving healthcare services, including maternal healthcare [12, 13]. It explicitly acknowledges power imbalances and biases that sustain barriers to culturally appropriate healthcare [11, 14] and requires inequalities to be challenged at both an individual and institutional level [14]. The practice of cultural humility promotes empathy and assists practitioners to acknowledge and respect cultural differences, being humble enough to ‘say that they do not know when they do not know’ [10, p. 119], and engage in ongoing self‐reflection to improve practice [15]. As such, it is a crucial step in providing high‐quality care to an increasingly diverse population within maternal health services [12, 16]. The approach goes beyond the idea of cultural competency which assumes the more knowledge a health professional has about another culture, the greater their level of competence will be in practice [13]. Tervalon and Murray‐Garcia [10] identified that cultural competency was both illusive and unattainable for health professionals. However, despite a growing focus on the concept, there remain knowledge gaps about how to foster cultural humility within healthcare settings [11, 13] and how to work in a culturally humble way [17].

## 2 Background

Australia is a multicultural society; 29.1% of its population was born overseas [18]. Governed by the Migration Act 1958, migration to Australia comprises of two main streams: (1) the refugee and humanitarian resettlement programme and (2) the migration programme. Quotas are applied to all streams with the humanitarian programme capped at 20,000 entrants per year with an additional 2000 per year for people who enter the country with a valid visa and the apply for refugee status [19]. The migration programme was capped at 190,000 for 2023–2024 [20]. Recent data has highlighted that approximately 25% of mothers who gave birth in Australia were born in countries where English is not the primary language [21].

In Australia, refugee and migrant women can experience racism, social deprivation, discrimination and a lower socioeconomic status resulting in cultural and language barriers to and within healthcare services including antenatal care [1, 22]. Some women are also vulnerable to trauma‐related mental health issues many years after their resettlement [23]. Pregnant women from these backgrounds can suffer from poor physical and mental health and long‐lasting impacts on their wellbeing requiring a greater need for culturally responsive healthcare [1]. Despite this potential need, they are also at risk of disparate healthcare access due to poor health literacy and difficulties navigating the healthcare system [24, 25]. In this context, these women are at higher risk of complications during childbirth and poorer pregnancy outcomes [4, 26, 27].

Furthermore, refugee and migrant women often have different health beliefs related to pregnancy and childbirth which are often not considered as part of their healthcare [28, 29]. For example, many refugee and migrant women do not view pregnancy as a medical condition and are more accustomed to community‐based care [30, 31]. Lack of cultural humility among health staff has been noted as a key concern of refugee and migrant women resulting in poor interpersonal relationships, discrimination, limited understanding of clinical interventions and negative experiences of the healthcare environment [12, 32]. Despite policies and standards for health professionals that emphasise the importance of culturally appropriate care [7, 33], health professionals can have little understanding of diverse perspectives related to pregnancy and childbirth and may base their practices on stereotypical assumptions about people from migrant and refugee backgrounds [9, 34].

The Australian healthcare system is decentralised, with both Federal and State policies shaping service provision. Maternal health services for refugee and migrant women are guided by policies focused on maternity care for all women (e.g. Pregnancy Care Guidelines) and some that are specific to refugee and migrant communities (e.g. NSW Plan for Healthy Culturally and Linguistically Diverse Communities 2019–2023; NSW Refugee Health Plan 2022–2027). Within this policy framework, maternal health services are expected to provide woman‐centred care that is responsive to and respectful of a woman's culture [7, 8, 35]. Yet service providers report being inadequately trained, insufficiently resourced and lacking confidence to deliver culturally responsive care [9]. The purpose of this study is to explore how to improve the experiences of refugee and migrant women with maternal health services through the lens of cultural humility. This study aimed to create an opportunity for stakeholders to engage in a dialogue to explore how best to teach the practice of cultural humility in healthcare settings and what a training package should look like.

## 3 Methods

### 3.1 Study Setting

The study was conducted in an Australian regional local health district with a substantial urban population and smaller towns and settlements. The percentage of the population born overseas is approximately 20% with 63% of this population speaking a language other than English at home [18]. The migrant and refugee populations differ considerably with recent migrant populations predominantly from India (19.2%) and China (9.5%) [18] and humanitarian populations mainly from the Democratic Republic of Congo, Syria, Iraq, Myanmar, Iran, Eritrea and Ethiopia [19].

The regional local health district has eight hospitals, the two largest offer antenatal services, an early assessment unit, a birthing unit, a midwifery‐led care unit and postnatal care (up to 6 weeks). Two other hospitals offer antenatal services through GP‐shared care and midwife outreach clinics located in the community. A maternity social work team also operates at hospitals with birthing units. Refugee and migrant‐specific health services in the area operate within the local health district.

### 3.2 Study Design

This research project used a World Café methodology to work with maternal health service providers and managers within a Local Health District (LHD), staff from the LHD's Multicultural and Refugee Health Service, and local refugee and migrant women, to discuss maternal healthcare in the region. A World Café methodology involves participants discussing a shared concern and working together to design potential solutions [36, 37]. World Cafés have been successfully used in collaborating with vulnerable communities including refugees and migrants [38, 39, 40]. In the context of this study, we adapted World Café design principles to support key attributes of cultural humility such as curiosity, openness, self‐reflection and compassion to build trust among all stakeholders [41], acknowledge lived experience and generate local knowledge and solutions [42].

For this study, we collaborated with the largest of the referral and training hospitals, the main multicultural and refugee healthcare provider in the region, and with refugee and migrant women who had used or currently use these services. Key stakeholders including maternal and multicultural healthcare providers were involved in all aspects of the research design including securing funding, developing the study aims and methods, ethics application and recruitment of participants.

The project team purposively recruited maternal healthcare providers such as midwives, nurses (antenatal and neonatal), physicians, social workers, birth educators and managers who work with women from refugee and/or migrant backgrounds. The multicultural health service personnel purposively recruited women who self‐identified as refugees and/or migrants from non‐English speaking backgrounds who had received maternal health services through the largest local hospital.

We ran two online sessions specially for refugee and migrant women to discuss the project, the process of the World Café including the consent procedure and to obtain their input on all aspects of the event. The World Café was held at the local city library, which is a central location and considered culturally safe by migrant and refugee community members. The day/time chosen took into consideration women's preferences and we provided childminding at the event for women with young children. Due to the diversity in participant's language backgrounds, we opted not to use interpreters as dialogue would need to be interpreted several times lengthening the time of the event. In consultation with stakeholders, we made the decision that women who required an interpreter would be better able to share their stories and voice their opinions in a small group interview with the support of an interpreter. We identified two groups of women (Arabic speaking and Burmese/Karen speaking) who required interpreters and conducted separate group interviews.

**Figure 1:**
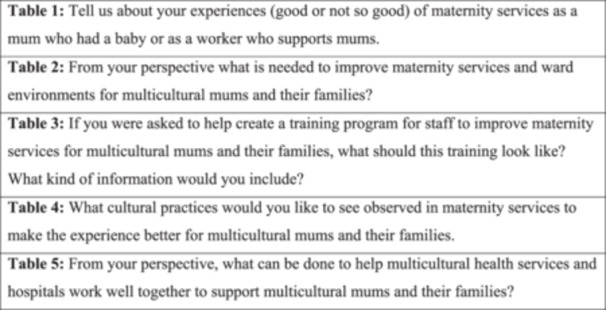
Cultural humility World Café questions.

The development of the topic guide involved several rounds of group dialogue with key stakeholders. We began the development of the topic guide by circulating a survey to determine maternal healthcare and multicultural healthcare providers' understanding of the term ‘cultural humility’, and the use of this and related terms within their organisations. Based on the responses, we decided that the topics for the World Café should draw on participants' experiences of maternal care and elicit their perceptions on how to improve care (see Figure 1).

### 3.3 World Café Process

The World Café involved mixed groups of maternal health service providers, multicultural healthcare providers and women from multicultural backgrounds who use these services. Participants sat at tables (*n* = 5) in groups of four or five per table. Each table focused on a different question (see Figure 1) for 20 min and then participants moved to the next table to share their ideas and expertise on another question. This process continued until all groups had rotated around the five tables. A member of the research team was stationed at each table to facilitate and audio record the group dialogue. The participants were also provided with stationery to take notes and to contribute anonymously using the suggestion box on each table. The World Café took approximately 4 h with two 10‐min breaks for refreshments.

The group interviews with Arabic‐speaking women and Burmese and Karenni women were held on separate days and locations of the World Café due to their need for interpreters. A bi‐cultural officer was present to ensure cultural safety and provide language support where needed. These interviews took approximately 1.5 h each. The interviews were audio‐recorded and only the English‐interpreted parts of the interviews transcribed.

All participants provided written consent for participation, and the Human Research Ethics Committee of the University of Wollongong and the local health districts of interest granted ethics approvals. Participants received a gift voucher to compensate them for their time.

### 3.4 Participants Characteristics

World Café participants (*n* = 34) included women from multicultural backgrounds (*n* = 20), maternal healthcare providers such as midwives, social workers and management (*n* = 5), multicultural healthcare providers (*n* = 7) and a community‐based birth educator (*n* = 1). The group represented a diverse range of cultural (*n* = 17) backgrounds including Azerbaijan, Brazil, Cambodia, China, India, Iraq, Japan, Lebanon, Libya, Myanmar, Pakistan, Palestine, Syria, Thailand, Togo, Türkiye and Vietnam.

Seven women participated in the group interviews: three from Arabic‐speaking backgrounds (Syria and Iraq) and four from Burmese and Karenni backgrounds. A member of the research team conducted the interviews in English and was supported by an interpreter. The composition of the sample is reflective of the refugee and migrant population in the local area and in Australia [18].

### 3.5 Data Analysis

All data (World Café, group interviews and field notes) were transcribed and analysed thematically [43]. Authors D.R.G., L.M., K.O. and J.W. read the transcripts and field notes for questions 1 and 5, 2, 3 and 4, respectively, and created major and minor codes for each question. The outcomes of these initial analyses were cross‐coded by the first author using QRS NVivo 12 software [44] to identify relationships and codes across the whole data set. These codes were reviewed by the research team and key themes were identified. To ensure rigour in our analysis we engaged with key stakeholders and participants to validate these themes. We invited participants to a member‐checking session to present findings to all stakeholders and receive their feedback [43]. The authorship team further refined themes based on feedback provided by stakeholders.

## 4 Results

The World Café provided an opportunity for refugee and migrant women and maternal healthcare providers to share experiences of receiving and providing care and ideas about how to improve the quality of care. We identified the following themes: (1) co‐design and co‐delivery of training with multicultural community members and health services: ‘We have to work together’, (2) understanding cultural nuances: ‘Culture is trustworthy’, (3) support before and after birth: ‘I'm not saying it's bad but there's no help’, (4) communication with women: ‘I was missing communication’ and (5) incorporating trauma‐informed care: ‘So much noise and so much going on’. A key finding was the need for staff training that is co‐designed and co‐delivered with members of multicultural communities and healthcare providers. The participants believed that training should include women's stories to capture cultural nuances around pregnancy and birthing, their support needs including the psychological and emotional ones, and the importance of effective cross‐cultural communication.

### 4.1 Co‐design and Co‐Delivery of Training With Multicultural Community Members and Health Services: ‘We Have to Work Together’

The co‐design and co‐delivery of training were identified as key ways to improve experiences for women from multicultural backgrounds. Participants recommended that training should ‘*involve real people and real cases*’ as opposed to textbook examples which may feel removed from providers' day‐to‐day practice. The ideal training was described as concrete and practical, avoiding abstract concepts and discussions. Participants suggested the training should be ‘*consumer‐driven*’ and centred on women's perspectives of care. As one provider summarised:

I think there should be training in different modalities of care, antenatally, from different cultures. And I think it should be consumer driven. So, if we're doing education for hypothetical, they say are midwives on different cultural practices, antenatally different ways of antenatal care and have it led by people from that specific culture.

(Healthcare professional)

Specifically, many of the conversations centred on cultural practices among refugee and migrant women. Women wanted to share these practices with healthcare providers and hoped that this knowledge would be spread through education and training. As one of the refugee women concluded:

For educating everyone; does it include from admin to midwives, doctors, nurses. Everyone has to be there. Everyone has to be on the same page.

(Healthcare professional)

In addition to the consumers' voices, participants reported the need for representatives from multicultural communities who work in the local healthcare settings to be involved in the development of training to make use of their expertise:

People of different culture, they work at the hospitals. 40% or 50% of people are actually people of culture in the hospital system. Doctors, nurses, so get them involved in, when you're designing those training programs.

(Healthcare professional)

Participants also recommended that training should be face‐to‐face and regularly scheduled to ensure ongoing professional development in working cross‐culturally. Specifically, rather than one‐off online training, which is what currently tends to be offered to staff in healthcare settings, ongoing support and discussions about issues related to working with refugee and migrant women are required. As one provider explained:

For the service providers, this is the training that I'm talking about – having a space that they can drop in literally for 30 min you know every 6 months or every 3 months, 30 min just go in that space and there's information and there's multicultural people around.

(Healthcare professional)

Learning how to work effectively across cultural differences was perceived as an ongoing process, that required frequent conversations and ongoing professional development.

Finally, in conjunction with the co‐design of training, participants recommended better collaboration and cooperation between mainstream and multicultural health providers. Improving cooperation between providers would facilitate ‘*more advocacy and more room for collaboration and networking*’. Specifically, practitioners suggested having regular meetings between key maternal health staff and multicultural health staff to discuss the needs of new or emerging migrant and refugee women or complex cases to improve women's pregnancy and birthing experience.

### 4.2 Understanding Cultural Nuances: ‘Culture Is Trustworthy’

Culture and cultural differences were identified as a crucial aspect of maternity health services for refugee and migrant women. Culture was discussed in terms of the cultural knowledge and practices around the perinatal period that some women bring with them, the risk of stereotyping refugee and migrant women, as well as some culturally inappropriate practices which women may face in mainstream services.

Refugee and migrant women shared a rich tapestry of cultural practices that were important to them during pregnancy and post‐birth including the use of massage, oils, wrapping, co‐sleeping, appropriate foods, naming and religious rituals. They often felt, however, that their cultural knowledge and expertise of pregnancy, birthing and childcare was dismissed by maternal healthcare providers:

There is a lot of knowledge in our community in terms of giving birth and care of the baby so perhaps when you are in maternity services and sometimes you feel like you're being patronised.

(Refugee/Migrant Woman)

They reported hiding or delaying many of these cultural practices, however, due to feeling judged or stigmatised by staff:

Being told that you're doing something strange, I think, is a big part…Or even worrying about being told that you're doing something strange.

(Refugee/Migrant Woman)

Women suggested that maternal health service providers simply ask about cultural approaches to birth and perinatal care that they would like observed. They acknowledged that some might not be possible to accommodate in an Australian clinical setting but being asked was a valued recognition of cultural difference that made them feel seen and respected.

Nevertheless, some participants believed maternity providers are at risk of stereotyping women from multicultural backgrounds. For example, they may make assumptions based on the woman's appearance, as explained by one of the multicultural health providers:

I know a lot of women in India wear scarf, hijab, and so, sometimes what happens, is these Indian women get given Arabic handout without even asking them. So, they speak completely different language, they will read Hindi, they will read Urdu, but they can't read Arabic, but they won't understand it.

(Healthcare professional)

To avoid stereotyping women, multicultural healthcare providers suggested that a ‘*cultural assessment*’ be included in antenatal appointments to document women's migrant history and ask about cultural practices that are important to the woman.

Women participants also voiced concerns about other harmful behaviours providers sometimes display especially related to feeding the baby;

When nurses are trying to teach you how to feed the baby which is important, but in my culture, that happens naturally, like the mother, grandmother, they all talk about feeding the baby right, so for me, if the nurse is… keeps on almost harassing me to feed the baby, it's a very difficult… communication breaks down at that point….

(Refugee/Migrant Woman)

Participants felt that providers placed a lot of pressure on them to breastfeed and did not take into consideration higher levels of gestational diabetes among some cultural groups or the impact of having a caesarean making women feel ‘unaccomplished’. The topic of male practitioners was brought up by participants on multiple occasions. In general, refugee and migrant women expressed a preference for female practitioners and interpreters due to the cultural inappropriateness of having a man present for examinations or during labour. Some preferred female practitioners as they felt more comfortable discussing female genital mutilation/cutting with another woman: ‘*I want a [much] female doctor because I have some things just female*’. When a female practitioner or interpreter was not available, they at least hoped for communication and ‘warning’ about who is going to see them, for example, to give them time to ‘*cover up*.’ However, some were never consulted about having a male practitioner:

After I gave birth, I had a second‐degree tear, and it was all women so I didn't really think about it and then a man came to do the suture and that was uncomfortable for me because I wasn't expecting it. I don't know why I wasn't expecting it you know, but it was quite a surprise. It wasn't really part of the discussion.

(Refugee/Migrant Woman)

Women would have felt more comfortable and prepared if the possibility of being attended by a male was discussed with them during the antenatal period.

### 4.3 Supports Before and After Birth: ‘I'm Not Saying It's Bad But There's No Help’

Participants discussed the need for more help and information related to practical, physical and emotional support. The support required changed from the antenatal period to the postnatal period. However, across both periods participants expressed that they needed more information about the level of support they could expect.

During the antenatal period, participants suggested the need for bilingual birth education classes that included practical information such as when to attend the hospital, where to go, and health literacy information such as appropriate birthing positions, what a contraction feels like, when to push and what happens immediately after the birth. The antenatal classes that some of them did attend were insufficient:

Even I did their classes, but it was my first time ever being in hospital. I never in my life been in a hospital, but overseas. When I went to the hospital, nothing like what they taught me in the program.

(Refugee/Migrant Woman)

Participants also reported that antenatal appointments often felt ‘*rushed*’ and women were asked many clinical questions and provided a lot of information. They did not, however, feel the information was always clearly explained or felt that they could ask questions:

I felt very much talked at. They give you a lot of information and a lot of big words and it's quite fast paced, and I just sat there, and I nodded.

(Refugee/Migrant Woman)

In addition, some participants found that having a different midwife for each appointment asking similar questions made the experience uncaring and diminished their trust in services.

Many participants reported the need for more physical and practical support during the postnatal period, especially while in the hospital. Practitioners commented on the high rates of induction and caesarean sections among refugee and migrant women. Many women also reported having an induction, assisted birth and/or caesarean. They seemed surprised by and unprepared for the little practical support they received, for example, with showering, changing and feeding the baby:

Like in my country when I had the caesarean first baby, there's two nurses helping me at home… but here I'm walking by myself.

(Refugee/Migrant Woman)

As many refugee and migrant women did not have family support available, they found the postpartum period, especially when recovering from a caesarean, particularly challenging.

Women also recommended the need for more emotional support. For example, one woman recalled the emotional difficulty she experienced postpartum:

I was so emotional. I was crying a lot. I would just lock the door on myself. I don't want to talk to anyone, but I didn't know I was in depression. Now I know.

(Refugee/Migrant Woman)

Not having an extended family nearby, and in some cases being completely alone exacerbated these symptoms:

Because when I came here, I had no one. In my background when everyone had a baby, everyone around her, they don't leave her, they don't. Here I didn't find that support.

(Refugee/Migrant Woman)

Participants emphasised that refugee and migrant women need to be given more information on postnatal depression in language and access to psychological support from a practitioner from their own cultural background who may also understand their migration history and trauma experienced.

The final area of support the women listed focused on education on caring for newborns. Participants believed that classes about what to expect in the baby's first year should be provided for first‐time refugee and migrant women before the baby is born. As one of them explained:

Do classes for the women that's going to be new mothers, the first child for them. Just explain them what they going to expect, how to look after your child and in a different, ages, how do you react when they cry, when they in the sickness, and, yeah, all this type of stuff.

(Refugee/Migrant Woman)

Women reported having no time or sufficient language skills to read the different booklets that were provided to them in the hospital after the baby was born. Furthermore, many women seemed unaware of local mothers' groups or were not referred to these.

Conversely, maternal healthcare workers felt that they could not adequately provide the support women needed due to organisational constraints, particularly staff shortages:

My colleagues and I, we did our best to tag team, but she didn't have a partner who could be there for 12 h…We didn't have the capacity to stay for 12 h and they wanted us to tag in and out and she ended up feeling very alone.

(Healthcare professional)

### 4.4 Communication Barriers: ‘I Was Missing Communication’

Communication barriers, both linguistic and cultural, were frequently discussed during the World Café. The refugee and migrant women believed that more and better communication from providers is required to ensure a more positive perinatal experience. Issues related to translating, interpreting, the pace of speech and cultural misunderstandings sparked a lot of debate.

The availability and use of interpreters appeared to be ad hoc rather than standard practice. Women reported that they were not always asked if they required an interpreter for appointments, and if this was not planned, an interpreter was often not available. Practitioners and women also spoke about challenges with interpreter use. For example, if women were late for the appointment the interpreter would not always be able to stay or conversely, if the interpreter was late women would not be able to stay for the whole appointment: ‘*Being a CALD [culturally and linguistically diverse] patient is challenging because we have to wait for interpreters…*’. Participants also believed that their birth experience would be improved if an interpreter were able to stay for the duration of the labour and birth even if they did not normally need this service. Women explained that while they were in labour it was difficult to process information and instructions in English:

I think the interpreters would help as well. Because at that time [labour], sometimes the barrier does matter, because we cannot listen, our languages we understand, the other language is not processed, we need to process in our language. So, it's more better that we need, we listen to in our language.

(Refugee/Migrant Woman)

Participants reported, however, that interpreters often only stayed for the signing of the consent documents. Sometimes the women did not need interpreters but just the pace to be slower. In general, participants found the communication they received in English was often delivered very quickly.

Participants also expressed the need for clearer communication and guidance and a consideration of cultural differences. Women felt that there was an assumption they knew what to expect and why they were being asked to do certain things:

I'm woman who's just had a baby and just had a surgery, the nurses pushing, always forcing me to the shower, there's a reason why she's doing it. I'm not opposing that. I'm just saying the communication of why that is being done.

(Refugee/Migrant Woman)

Practitioners agreed that ideally women should have conversations with them before labour to discuss a birth plan and their options if the birth plan cannot be followed for clinical reasons,

So that says so much for women knowing what choices you have and opportunities there are before you're in labour, rather than having to be in that situation and make the decisions on the spot and you're just relying on clinicians to make those decisions for you.

(Healthcare professional)

Engaging in conversations about birth plans and options if the birth plan cannot be followed during the antenatal period may avoid some of the communication issues during labour and birth.

Some participants also mentioned cultural differences in how providers tend to communicate and what refugee and migrant women may require. Women expressed a preference for a more direct form of communication, especially when they were unsure about what to do. As one woman explained, using a less direct communication style can result in confusion:

Are you saying, can I give baby bottle, or not? Just tell me. ‘On the first day, we really don't want you to have the bottle milk, we want you to have breastmilk'. That's easy, simple to understand, you know, I'm not stressing about it then, I'm just like, “Okay.” Then they, but the communication from them would be like, “It's your choice, however, we are going to tell you that every, we're going to explain it to you that breastmilk is the best,” but then they've got this whole story, and I'm lost already. And I'm like, almost flower talking, I'm like, “Okay well, yeah, that means I'm confused, I don't know what to do.

(Refugee/Migrant Woman)

Participants thus wanted more guidance and direct communication from staff on mothering, particularly in the context of their new country.

### 4.5 Incorporating Trauma‐Informed Care: ‘So Much Noise and So Much Going On’

Finally, participants, specifically practitioners, discussed the importance of adopting a trauma‐informed care approach when working with refugee and migrant women. They believed that multicultural training needs to include the principles of trauma‐informed care to improve understanding of complex experiences of trauma. As one provider concluded:

There could be an extra layer of already‐accumulated trauma in their lives, especially even just from having to relocate their families and be pregnant, and that I think that there should be kind of a particular care around that.

(Healthcare professional)

Women also identified the impact of the trauma they had experienced on their ability to engage with practitioners in a meaningful way and the implications this had for their care. One woman summarised her experience in the following way:

I remember every time I get to that appointment, there were so many ladies and so much noise and so much going on and it's so triggering for me that my mind goes into hyper arousal state where it's not even me. And there'd be so many times that they gave me papers that said that I'm to pay bills and I could barely see but I smile all the time so they thought I was ok with it. And then next appointment, “But we told you that last week.” I wasn't here. But then on my record, my record shows my experience to indicate that I had some level of trauma or whatever to help guide that conversation. So, you know, you should be culturally awake.

(Refugee/Migrant Woman)

The importance of paying attention to refugee and migrant women's psychological and emotional needs especially if they have had prior experiences of trauma, should be incorporated into the multicultural training for providers.

## 5 Discussion

This study makes a significant contribution to the field of maternal health for refugee and migrant women and multicultural health more broadly. Using cultural humility as a lens combined with the World Café methodology to explore ways to improve experiences of maternal health services for refugee and migrant women is new in Australia. This combination provided participants with an opportunity to work towards a solution to some of the issues experienced by refugee and migrant women when engaging with maternal health services.

Broadly, we found that maternal health services may not acknowledge or accommodate important cultural beliefs and practices of refugee and migrant women. Some women did acknowledge the individual efforts of practitioners to support them during their pregnancy and birth. However, the study highlighted several potential improvements to the provision of maternal healthcare to refugee and migrant women during the perinatal period. Specifically, it indicated the need to co‐design multicultural training for all maternal health staff with the aim of (1) increasing understanding of cultural factors around peri‐post‐natal care, (2) creating awareness of refugee and migrant women's maternity support needs, (3) clearer communication and guidance and (4) an inclusion of trauma‐informed care. The training needs to be co‐delivered with refugees and migrant women and occur regularly and face‐to‐face to sustain strong connections. This approach embraces the principles of cultural humility, which recognises that individuals are experts in their own experiences and requires practitioners to engage with compassionate curiosity and a genuine desire to understand the individual [14]. Being culturally humble also acknowledges the power differentials that exist between not only the practitioner and women but also between the culture and experiences of the refugee and migrant woman and the dominant culture of the broader society [45, 46]. Healthcare providers must show a genuine interest in understanding refugee and migrant women's experiences, and how these experiences shape who they are and the care they need. Finally, co‐designing and co‐delivering training to maternal healthcare providers requires an institutional commitment and accountability to ensure cultural humility [10]. Healthcare institutions need to develop and sustain connections with refugee and migrant women and invite them into the decision‐making on how to improve the services. Ultimately, to ensure engagement in and sustainability of these initiatives, both bottom‐up and top‐down commitment is required.

The study also found that information about maternal healthcare options and procedures is often not adequately provided to migrant and refugee women due to linguistic and cultural communication barriers. Consequently, women are less likely to understand information provided by practitioners or to be kept informed particularly during labour and birth. This is consistent with [31] who reported that migrant and refugee women are less likely to know they have a right to be involved in maternal health decisions, leaving them vulnerable to information gatekeeping and paternalistic approaches by healthcare providers. Efforts to improve communication with refugee and migrant women are therefore essential to the provision of culturally humble care. Providers should speak clearly, avoid local slang, and frequently check for understanding, for example, by using the teach‐back method which asks women to recall and restate what they have been told [47]. This and other health communication tools are not a test of the women's knowledge but a test of how well the healthcare provider explained the concept and provided an opportunity for the woman to effectively consent and choose.

Ensuring good communication between practitioner and refugee and migrant women also often means engaging an interpreter. In Australia, healthcare interpreters are available face‐to‐face or by telephone in healthcare services [48]. Professionals can also access the free Translating and Interpreting Service (TIS) which employs accredited interpreters. TIS offers telephone interpreting service 24 h a day, 7 days per week [49]. Our study showed that when providers are assessing the need for an interpreter it is recommended to err on the side of caution and provide an interpreter. ‐Women may appear to have sufficient English proficiency for everyday social engagement but insufficient English to understand technical terms, medical terminology and procedures especially in highly stressful situations such as childbirth. Even when complex terminology is not being used, health concepts require a sophisticated understanding of language, especially if the person is to have the opportunity to interrogate the information adequately and be able to make an informed decision [8, 50]. When requesting an interpreter, practitioners should consider the woman's preferred language, ethnicity, religion and dialect, as well as their preference for the gender of the interpreter [50]. Healthcare providers should avoid using family members including partners or children and friends as they may not be able to interpret complex medical terms or procedures, and women may feel uncomfortable disclosing information in their presence [7, 51]. It may not be possible to accommodate all individual preferences regarding interpreter requirements. However, understanding women's concerns and informing them of available options, while clarifying the role of interpreters as facilitators of communication who are bound by confidentiality and impartiality, helps build trust and effective partnerships.

Finally, consistent with previous studies [31, 52], the findings highlighted the need to incorporate trauma‐informed care into maternity services with refugee and migrant women. Trauma‐informed health services recognise how trauma affects people's lives, their healthcare needs and their experiences in health services and respond by integrating knowledge about trauma into policies and practices [53, 54]. Within maternal healthcare, trauma‐informed care and cultural humility can offer the possibility of health services experiences where people feel both supported and validated [53, 55]. The participants' experiences identified in this research highlight that understanding the impact of trauma can allow for more effective support and nurture deeper interpersonal connections between the healthcare provider and woman [55, 56]. In combination, cultural humility and trauma‐informed care provide the basis for the delivery of health services that is respectful for women's life experiences and cultural beliefs.

### 5.1 Limitations and Future Research

Given the exploratory nature of the study, the purposive sampling methods, and the focus on one region in Australia only, we should avoid generalising from these findings. It is also likely that the experiences of other professionals working with refugee and migrant women, such as general practitioners or obstetricians, may vary.

Given these limitations and the gaps in research on the translation of cultural humility principles to practice, there is ample room for future studies including an examination of the individual and organisational efforts in maternal health services to enhance the quality of care of refugees and migrant women. Future research should also document the process and the outcomes of co‐designing and co‐delivering cultural humility training for maternal healthcare providers with refugee and migrant women.

## 6 Conclusion

By bringing refugee and migrant women and the health providers together to discuss their lived experiences on both ends of these cross‐cultural encounters, this World Café event was a first step towards multicultural communities and healthcare institutions in the region working together to enhance cultural humility within maternal health services. It allowed each group to hear the perspectives and experiences of the other, build rapport and open a needed dialogue on how to generate local knowledge and local solutions. All participants agreed on the need for better training for healthcare providers and for it to be co‐designed and co‐delivered to refugee and migrant women. The opportunities for providers and women to truly listen to each other and work together demonstrate that cultural humility is more than an abstract concept but an embodiment of all that it stands for.

## Author Contributions

**Delia Rambaldini‐Gooding:** conceptualisation, investigation, funding acquisition, writing–original draft, writing–review and editing, project administration, supervision, methodology, data curation, formal analysis. **Katarzyna Olcoń:** conceptualisation, investigation, writing–original draft, funding acquisition, writing–review and editing, methodology, formal analysis. **Luke Molloy:** conceptualisation, investigation, funding acquisition, writing–original draft, methodology, writing–review and editing, formal analysis. **Leissa Pitts:** writing–review and editing, conceptualisation, methodology, resources, validation. **Sofia Lema:** conceptualisation, methodology, writing–review and editing, resources, validation. **Eman Baghdadi:** writing–review and editing, resources, validation. **Jane Williams:** formal analysis, investigation, writing–review and editing. **Chris Degeling:** conceptualisation, funding acquisition, methodology, writing–review and editing, investigation.

## Ethics Statement

Our study was approved by the joint University of Wollongong & Illawarra Shoalhaven Local Health District Health and Medical Human Research Ethics Committee (approval no. 2024/ETH01009).

## Consent

All participants were provided with a participant information sheet and provided written informed consent before enrolment in the study.

## Conflicts of Interest

The authors whose names are listed immediately below certify that they have no conflicts of interest including, but not limited to, patent or stock ownership, membership of a company of board of directors, membership of an advisory board or committee for a company and consultancy for or receipt of speaker's fees from a company.

## Data Availability

The deidentified data that support the findings of this study are available from the corresponding author upon reasonable request. To protect participants confidentiality and privacy, UOW Research Data Storage Policy states that all data must be securely stored, and we have included this in our Participant Information Sheet.
